# The effects of ghrelin and LEAP-2 in energy homeostasis are modulated by thermoneutrality, high-fat diet and aging

**DOI:** 10.1007/s40618-024-02307-4

**Published:** 2024-02-09

**Authors:** S. Casado, M. Varela-Miguéns, T. de Oliveira Diz, C. Quintela-Vilariño, R. Nogueiras, C. Diéguez, S. Tovar

**Affiliations:** 1 Departamento de Fisioloxía and Centro de Investigación en Medicina Molecular (CIMUS), Universidade de Santiago de Compostela, Instituto de Investigaciones Sanitarias de Santiago de Compostela (IDIS), 15782 Santiago de Compostela, Spain; 2 CIBER Fisiopatología de la Obesidad y Nutrición (CIBERobn), 28029 Madrid, Spain

**Keywords:** Ghrelin, LEAP-2, Energy, Obesity, Aging, Adipose tissue

## Abstract

**Purpose:**

Liver-expressed antimicrobial peptide 2 (LEAP-2) has been recently identified as the endogenous non-competitive allosteric antagonist of the growth hormone secretagogue receptor 1a (GHSR1a). In rodents, LEAP-2 blunts ghrelin-induced feeding and its plasma levels are modulated in response to nutritional status, being decreased upon fasting and increased in high-fat diet (HFD) fed mice. Clinical data support the regulation of circulating LEAP-2 by nutrient availability in humans. In this work, our primary objective was to examine the chronic effects of ghrelin and LEAP-2 administration on food intake, adiposity, and energy expenditure in young mice subjected to standard and HFD at both room temperature and at thermoneutrality. Furthermore, we aimed to assess the impact of these two hormones on aging mice.

**Results:**

Our results indicate that LEAP-2 produces a significant decrease of body weight and adiposity, an increase in energy expenditure, and activation of the thermogenic program in white and brown adipose tissue depots. However, this effect is not maintained under HFD or under thermoneutral conditions and is only partially observed in aging mice.

**Conclusion:**

In summary our studies describe the central effects of LEAP-2 within distinct experimental contexts, and contribute to the comprehension of LEAP-2's role in energy metabolism.

**Supplementary Information:**

The online version contains supplementary material available at 10.1007/s40618-024-02307-4.

## Introduction

It is well established that the ghrelin system plays a key role in energy homeostasis and the evidence are as follows. Ghrelin administration elicits a marked increase in adiposity in rodents [1] and a potent orexigenic effect in almost all species tested, including humans [2, 3]. This orexigenic effect is specifically mediated by acylated ghrelin, which acts through specific hypothalamic receptors known as GHSR1a [4–6]. These receptors operate via well-defined signaling pathways that have been recently elucidated [7–10]. It is noteworthy that, GHSR1a can form heterodimeric complexes with receptors such dopamine 1 receptor (D1R), dopamine 2 receptor (D2R) and melanocortin-3-receptor (MC3R), all of which are implicated in the regulation of energy homeostasis [11–13].

The discovery of liver-expressed antimicrobial peptide 2 (LEAP-2), as a recently identified endogenous non-competitive allosteric antagonist of the GHSR1a, stands out as the most unexpected finding in the field related to the ghrelin system [14, 15]. LEAP-2 blunts ghrelin-induced intracellular calcium mobilization in vitro and ghrelin-induced GH secretion and food intake in vivo [14]. Interestingly, in relation to energy balance, circulating levels of ghrelin and LEAP-2 behave in opposite fashion in response to feeding and fasting [16].

The discovery of LEAP-2 as a new component of the ghrelin system is considered a major breakthrough, offering potential insights into the role of the ghrelin system in energy and metabolic homeostasis and paving the way for the development of more rational therapies. Recent data have shown that treatment of obesity is feasible through a unimolecular polypharmacology, which involves simultaneously targeting multiple receptors [17–19]. Whether blocking GHSR1a could further enhance this approach is unknown. However, before reaching this stage, it is widely acknowledged that more preclinical data are needed to assess the influence of factors such as diet, temperature and age, all of which may variably affect the biological effects of ghrelin and LEAP-2 on energy homeostasis [11–13, 20].

In this study, our objective was to investigate the impact of intracerebroventricular chronic administration of ghrelin and LEAP-2 on food intake, adiposity, and energy expenditure. It has been described that ghrelin’s central effects on nutrient intake and nutrient partitioning can be separated following exposure to HFD, indicating that they act through independent neuronal pathways [21]. Therefore, we conducted these experiments in mice that were maintained on either a standard diet or a high-fat diet. While the majority of mouse experiments are typically conducted at 22–24 °C, [22], a temperature well below their thermoneutrality zone, we also conducted an experiment at 30 °C to explore potential temperature-related effects. Furthermore, recognizing the age-dependent nature of ghrelin's biological effects on energy homeostasis, we conducted a specific study focusing on older mice.

## Materials and methods

### Animals

C57BL6j mice were housed in air-conditioned rooms (22–24 °C) under a 12:12 h light/dark cycle. After weaning, the mice were fed with standard chow (STD) or the high fat diet (HFD) (Research Diets D12,492; 60% fat, 5.24 kcal g^−1^, Research Diets, New Brunswick, NJ) for 15 weeks.

For aging experiments other group was maintained until 30 months of age.

After the experiment, the mice were sacrificed, and all tissues were rapidly explanted and snap frozen on dry ice. The tissues were collected and frozen at − 80 °C until they were used.

The animal procedures were conducted according to the principles approved by the Animal Care Research Bioethics Committee from University of Santiago de Compostela (license 15010/17/002); performed in compliance with the Directive 2010/63/EU and Spanish Royal Decree 53/2013, on the protection of animals used for experimental and other scientific purposes.

### Intracerebroventricular (icv) chronic treatment of ghrelin and/or LEAP-2 on food intake and body weight

Brain infusion cannulas were stereotaxically placed into the lateral ventricle as previously described [23]. A catheter tube was connected from the brain infusion cannula to an osmotic minipump flow moderator (model 1002D, Alza Corp., Palo Alto, CA). Mice were then infused with either vehicle or ghrelin (Bachem) (5 nmol/day) or LEAP-2 (LEAP-2(38–77) peptide (Biorbyt) (2 nmol/day) or ghrelin + LEAP-2 for 7 days into the lateral ventricle as described previously [24, 25]. During this time, mice were motorized in body weight and food intake. The dose of ghrelin and LEAP-2 was chosen based on our experiments or a previous study [26–28].

### Determination of energy balance

The animals were monitored in a custom 12-cage indirect calorimetry-, food intake-, and locomotor activity-monitoring system (LabMaster; TSE Systems; Bad Homburg, Germany) as described elsewhere [23].

For thermoneutrality experiments the mice were maintained to thermoneutral environment (30 °C with relative humidity of 45–52% in cages in LabMaster; TSE Systems; Bad Homburg, Germany).

For cold exposure and thermoneutrality***,*** mice were fasted for 1 h before they were placed in a room at 4 °C. We measured the core body temperature at various time points before and during cold exposure using a rectal probe attached to a digital thermometer (Harvard Apparatus, Holliston, MA, USA).

### Hormonal and biochemical assays

The levels of various compounds in plasma were measured by ELISAs. T_4_ (DRG, Germany), intra-assay and inter-assay variation coefficients was < 5% and < 8%, respectively. The assay sensitivity limit was 8 nmol/L, IGF1 (R&D Systems, USA) intra-assay and inter-assay variation coefficients was < 6% and < 10%, respectively. The assay sensitivity limit was 8.4 pg/mL, Leptin (Merk-Millipore) intra-assay and inter-assay variation coefficients was < 2% and < 5%, respectively. The assay sensitivity limit was 0.05 ng/mL and LEAP-2 (Human LEAP-2 [38–77] ELISA kit, Phoenix Pharmaceuticals, Inc) as described previously [29, 30], intra-assay and inter- assay variation coefficients was < 10% and < 15%, respectively. The assay sensitivity limit was 0.15 ng/ml. Triglycerides and cholesterol plasma levels were quantified by a homogeneous enzymatic colorimetric assay (Spinreact, S.A., Spain).

### Haematoxylin–eosin staining and immunohistochemistry

Sections of 3 μm thick were made on a microtome and stained by the standard haematoxylin–eosin alcoholic procedure according to the manufacturer’s instructions (BioOptica, Italy). Immunohistochemical analysis was performed with an anti-UCP1 antibody (1:1000 dilution) (cat. #ab10983; Abcam, USA) as described [30].

### Western blot analysis

Western blots were performed as previously described [25, 31] with antibodies against PPARγ (sc-7273) (Santa Cruz Biotechnology, USA); β_3_-AdR (ab694506), FGF21 (ab171941), PRDM16 (ab106410) and UCP1 (ab10983), FAS (ab128870), ACC **(**ab45174) (Abcam, USA); vinculin (clone hVIN-1) (Sigma-Aldrich cat# V9131); GAPDH (CB1001) (Millipore, USA). The protein levels were normalized to GAPDH or vinculin for each sample.

### Quantitative RT-PCR analysis

Real-time PCR (TaqMan; Applied Biosystems, USA) was performed with specific primers and probes as previously described [32, 33]. Relative values were expressed in relation to hypoxanthine–guanine phosphoribosyl-transferase (*Hprt, Mm03024075_m1*) levels. We used the following commercially available and pre-validated TaqMan primer/probe sets (Applied Biosystems) for leap2 (Mm 00461982_g1).

### Statistical analysis

Values were plotted as the mean ± SEM for each genotype. To test whether the data follows a Gaussian distribution, a normality test was performed (the Kolmogorov–Smirnov test for n between 5 and 7 or the Shapiro–Wilk test for n > 7). For normal distributions, a parametric test was conducted; for two‐group comparisons, the unpaired t‐test was carried out. In the statistical analysis data with a p‐value less than 0.05 were considered statistically significant. For a multiple comparison test, a one‐way ANOVA followed by Bonferroni´s post hoc multiple comparison test was performed. Data analysis was performed in GraphPad Prism Software Version 9.0 (GraphPad Software San Diego, CA, USA).

## Results

### Effect of chronic central infusion of LEAP-2 in STD diet on body weight, food intake adiposity and energy expenditure

**Fig. 1:**
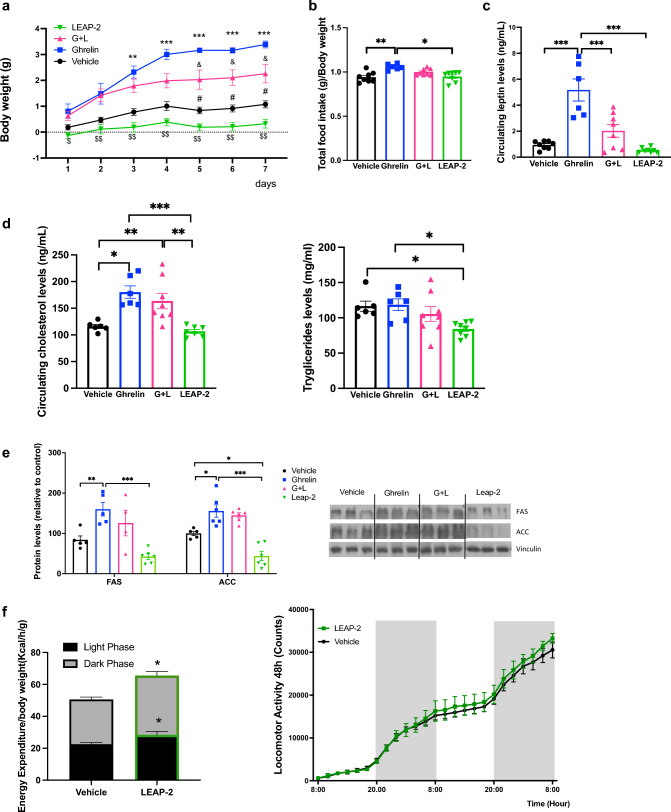
Central chronic LEAP-2 on a STD diet decreased body weight and increase energy expenditure. **a** Body weight change in mice with administration of LEAP-2, ghrelin, ghrelin and LEAP-2 or vehicle during 7 days. Data is expressed as mean ± SEM *p ≤ 0.05, **p ≤ 0.01, ***p ≤ 0.001 vehicle vs ghrelin; **#**p < 0.05, LEAP-2 vs vehicle; $p < 0.05, $$p < 0.01 LEAP-2 vs ghrelin + LEAP-2; &p < 0.05 ghrelin vs ghrelin + LEAP-2. **b** Accumulated food intake normalized to body weight during all treatment. **c** Circulating levels of leptin in all groups after 7 days of treatment. **d** Circulating cholesterol and triglycerides levels after 7 days of treatment. **e** Semiquantification of immunoblot analysis of de novo lipogenesis proteins FAS and ACC in WAT and representative immunoblot after 7 days treatment. **f** Energy expenditure corrected by body weight and locomotor activity during 48 h in control and LEAP-2 treated mice. Data is expressed as mean ± SEM *p ≤ 0.05, **p ≤ 0.01, ***p ≤ 0.001

As anticipated, ghrelin administration led to a notable increase in body weight compared to the vehicle-treated group. In contrast, mice treated with LEAP-2 exhibited a reduced weight gain (Fig. 1a). The co-administration of LEAP-2 with ghrelin attenuated the ghrelin-induced increase in body weight an effect that was independent of food intake (Fig. 1a, b). Food intake in LEAP-2 treated animals remained unaltered relative to controls, whereas it increased in ghrelin-treated animals. Circulating leptin levels, a surrogate marker of adiposity, remained unchanged in LEAP-2 treated animals compared to controls, while it suppressed the ghrelin-induced increase in leptin levels (Fig. 1c). Additionally, LEAP-2 administration led to a reduction in circulating triglyceride levels, while cholesterol levels remained unaffected (Fig. 1d), but co-administration of LEAP-2 with ghrelin failed to reverse the ghrelin-induced increase in cholesterol (Fig. 1d).

In the white adipose tissue (WAT) LEAP-2 decrease fatty acid synthase (FAS), and acetyl CoA carboxylase 1 (ACC1) compared with control animals with statistically differences in ACC and clear tendency in FAS. However, LEAP-2 failed to reverse the ghrelin action in the expression of these enzymes when the LEAP-2 and the ghrelin are co-administered (Fig. 1e).

Because LEAP-2 administration reduced the body weight compared with control mice and to uncover the underlying mechanism responsible for the reduced body weight and adiposity, we assessed energy expenditure through indirect calorimetry in both control and LEAP-2 treated mice. This analysis revealed a significant increase in energy expenditure in mice treated with LEAP-2, evident in both light and dark phases, and this effect was not linked to changes in locomotor activity (Fig. 1f).

### Chronic central infusion of LEAP-2 in STD diet-fed mice increases thermogenic markers in BAT and browning in scWAT.

**Fig. 2:**
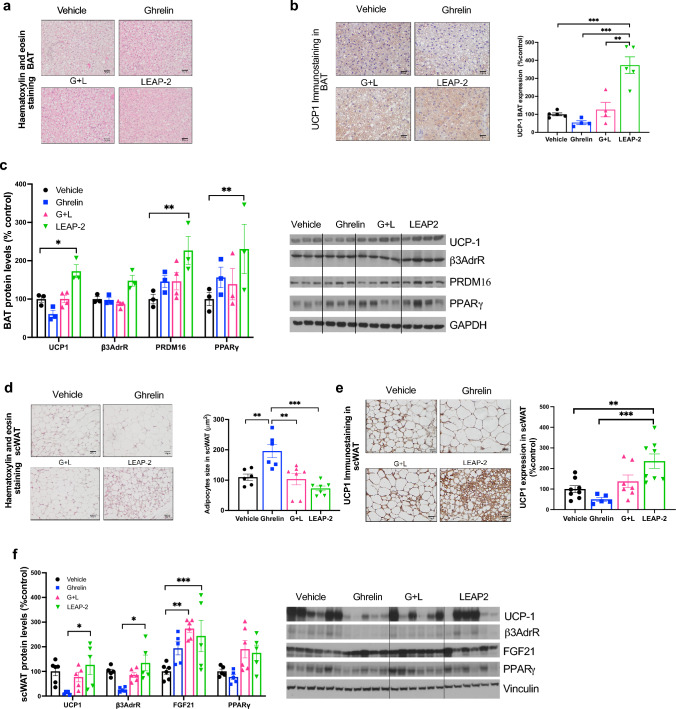
Central chronic LEAP-2 on a STD diet increase thermogenic markers in BAT and browning in scWAT. **a** Representative Hematoxylin & Eosin staining of BAT of treated mice after 7 days of treatment. **b** Representative UCP1 immunohistochemistry and semiquantification of BAT after 7 days treatment. **c** Semiquantification of immunoblot analysis of thermogenesis proteins as UCP1, β3-adrenergic R, PRDM16, PPARγ in BAT and representative immunoblot. **d** Representative Hematoxylin & Eosin staining and size measurements of scWAT. **e** UCP1 immunohistochemistry of scWAT and semiquantification of UCP1. **f** Quantification of immunoblot analysis of thermogenesis proteins as UCP1, β3-adrenergic R, FGF21 and PPARγ in scWAT and representative immunoblot. Data is expressed as mean ± SEM. *p ≤ 0.05, **p ≤ 0.01, ***p ≤ 0.001

We delved into the molecular mechanisms underlying the observed increase in energy expenditure. In line with the lower body weight and heightened energy expenditure in LEAP-2-treated mice, histomorphological analysis of brown adipose tissue (BAT) revealed smaller lipid droplets (Fig. 2a). Immunohistochemistry showed elevated  uncoupling protein 1 (UCP1) levels in mice treated with LEAP- 2 (Fig. 2b). This result was corroborated by Western blot (WB) analysis, indicating increased protein levels of various thermogenic biomarkers, including UCP1, peroxisome proliferator-activated receptor gamma (PPARγ), and PR domain zinc finger protein 16 (PRDM16), indicative of heightened thermogenic activity (Fig. 2c).

Subsequently, we investigated the subcutaneous white adipose tissue (scWAT). Ghrelin-treated animals exhibited expanded adipose tissue size, which was partially ameliorated when co-administered with LEAP-2 (Fig. 2d). This alteration mirrored characteristics commonly associated with brown adipose tissue (BAT) development, as corroborated by elevated UCP1 expression observed through immunostaining in LEAP-2-treated animals (Fig. 2e). Additionally, LEAP-2 administration induced a rise in β3-adrenergic receptor (β3AdrR) expression, but not UCP1, compared to controls. Notably, ghrelin displayed a non-statistically significant trend towards reduced β3AdrR expression. Moreover, LEAP-2 administration elicited a pronounced surge in fibroblast growth factor 21 (FGF21) expression (Fig. 2f).

In sum, LEAP-2 can influence energy balance, under normal dietary and housing conditions, through an enhancement of energy expenditure which is explained by increased BAT thermogenic activity and the browning of scWAT, a mechanism independent of thyroids hormones (Fig S1a).

### Effect of chronic central infusion of LEAP-2 in Energy Homeostasis under Thermoneutral Conditions and cold exposure

We evaluated the effect of LEAP-2 treatment in a thermoneutral environment (30 °C) to eliminate the extra metabolism necessary to maintain body temperature at lower ambient temperatures.

**Fig. 3:**
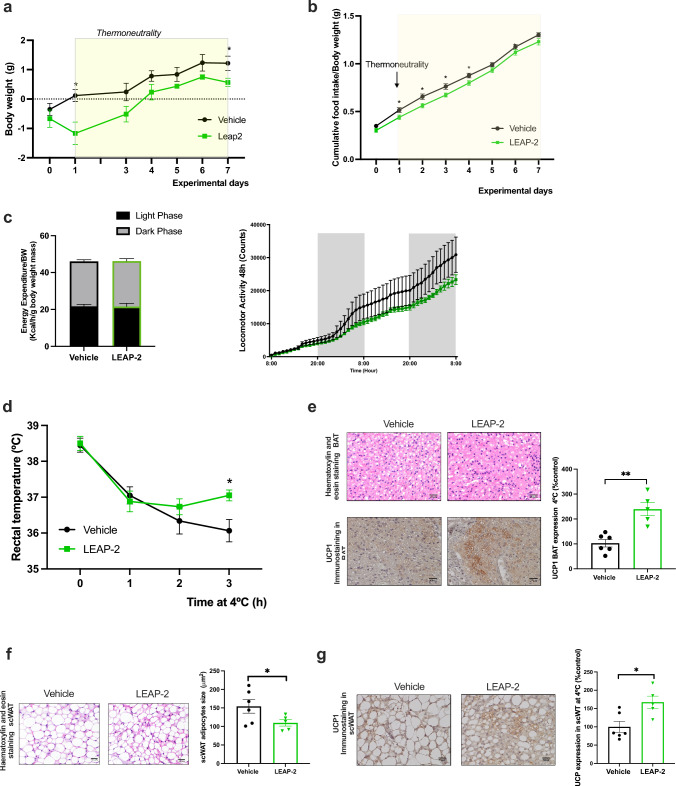
Effect of chronic central infusion of LEAP-2 in Energy Homeostasis under Thermoneutral Conditions and cold exposure. **a** Body weight change in mice with administration of LEAP-2 or vehicle during 7 days in thermoneutrality starting at day 1. **b** Accumulated food intake in relation of body weight during thermoneutrality. **c** Energy expenditure corrected by body weight and locomotor activity during 48 h in control and LEAP-2 treated mice. **d** Rectal temperature of mice treated with LEAP-2 or vehicle upon cold exposure (+ 4 °C) for 3 h. **e** Representative Hematoxylin & Eosin staining of BAT and representative UCP1 immunohistochemistry of treated mice after 3 h of 4ªC and semiquantification of UCP1 immunohistochemistry of BAT. **f** Representative Hematoxylin & Eosin staining of scWAT and scWAT adipocyte size of treated mice after 3 h of 4ªC. **g** Representative UCP1 immunohistochemistry and semiquantification of UCP1 immunohistochemistry of scWAT. Data is expressed as mean ± SEM *p ≤ 0.05, **p ≤ 0.01, ***p ≤ 0.001 vehicle vs LEAP-2

A marginal, significant decrease in body weight gain was observed at the end of the experiment (day 7) (Fig. 3a). However, no significant differences were observed in final body weights (Fig. 3a) or food intake (Fig. 3b) in mice treated with LEAP-2 compared to controls. Of note, under thermoneutral conditions, energy expenditure and locomotor activity did not change with the treatment (Fig. 3c). These results indicate that chronic central infusion of LEAP-2 failed to exert a major impact on body weight, with significance observed only at day 7, and even then, the effects were less pronounced than those observed at 22ºC.

To gain deeper insight into the physiological relevance of LEAP-2 during cold adaptation, we conducted a cold tolerance test. At the 3-h mark of cold exposure, LEAP-2 treated mice appeared to better maintain their body temperature compared to controls (Fig. 3d). Histological analysis further revealed smaller lipid droplets in the BAT of LEAP-2 group, accompanied by a significant up-regulation of UCP1 protein as observed in immunostaining (Fig. 3e). Additionally, we conducted a histomorphological analysis of scWAT for study browning, which demonstrated a reduction in adipocyte size in LEAP-2-treated mice (Fig. 3f). Consistently, UCP1 immunostaining was increased in LEAP-2-treated animals compared to those in the control group (Fig. 3g). Altogether, these findings could suggest that LEAP-2 would improve the cold tolerance, at least partially, by increasing the browning of scWAT and the activation of BAT.

### HFD fed mice are resistant to the weight reduction of central LEAP-2

**Fig. 4:**
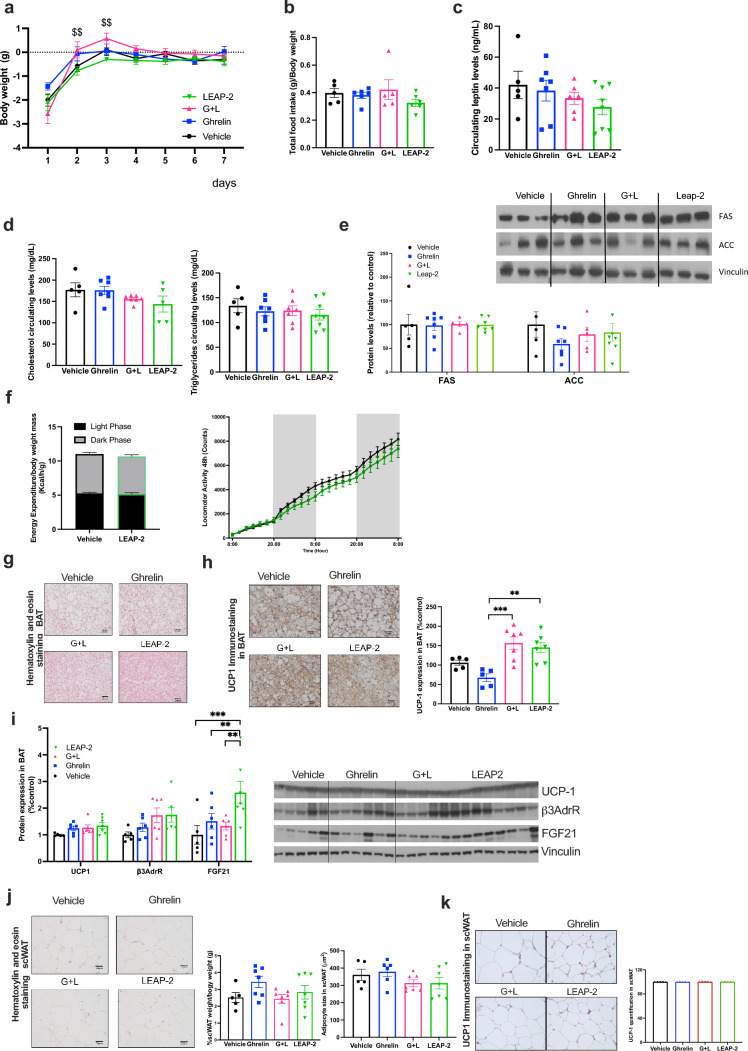
LEAP-2 Resistance in Energy Homeostasis and Thermogenesis Program in Mice on a HFD. **a** Body weight change in mice on a HFD with administration of LEAP-2, ghrelin, ghrelin and LEAP-2 or vehicle during 7 days. **b** Accumulated food intake in relation of body weight during all treatment. **c** Circulating levels of leptin in all groups after 7 days of treatment. **d** Circulating cholesterol and triglycerides levels after 7 days of treatment. **e** Quantification of immunoblot analysis of de novo lipogenesis proteins FAS and ACC in WAT and representative immunoblot after 7 days treatment. **f** Energy expenditure corrected by body weight and locomotor activity during 48 h in control and LEAP-2 treated mice under HFD. Data is expressed as mean ± SEM #p < 0.05, ##p < 0.01, ###p ≤ 0.001 ghrelin vs ghrelin + LEAP-2; $p < 0.05, $$p < 0.01, $$$p ≤ 0. LEAP-2 vs ghrelin + LEAP-2. **g** Representative Hematoxylin & Eosin staining of BAT of treated mice after 7 days of treatment in mice on a HFD. **h** Representative UCP1 immunohistochemistry and semiquantification of UCP1 of BAT after 7 days treatment. **i** Quantification of immunoblot analysis of thermogenesis proteins as UCP1, β3-adrenergic R and FGF21 in BAT and representative immunoblot. **j** Representative Hematoxylin & Eosin staining, weight and adipocyte size measurements of scWAT. **k** Representative UCP1 immunohistochemistry and semiquantification of UCP1 of scWAT. Data is expressed as mean ± SEM. *p ≤ 0.05, **p ≤ 0.01, ***p ≤ 0.001

As expected, our data indicated an increase in both circulating and liver mRNA expression of LEAP-2 in mice exposed to HFD for 12 weeks (Fig. S1c, d). Therefore, we decided to investigate the effect of chronic central infusion of LEAP-2 in diet-induced obese animals. In contrast to the data obtained in animals under standard diet (STD), we did not observe any difference in body weight gain (Fig. 4a), food intake (Fig. 4b) or adiposity as assessed by circulating leptin levels (Fig. 4c) between any of the treatments. Consistent with these findings, there were no changes in circulating levels of triglycerides or cholesterol among all the groups (Fig. 4d), and we also failed to detect changes in protein levels of FAS and ACC1 in WAT (Fig. 4e) or in energy expenditure and locomotor activity (Fig. 4f) in LEAP-2 treated mice.

As we expected, the histomorphological analysis revealed no major differences in BAT morphology (Fig. 4g) or UCP1 levels by immunohistochemistry (Fig. 4h) or Western blot (Fig. 4i). In relation of browning, we did not find any differences in scWAT histomorphology, adipocyte size (Fig. 4j) or UCP1 staining (Fig. 4k), indicating that under HFD, the mice exhibit resistance to the actions of LEAP-2. Overall, these findings confirm the resistance to adiposity actions for both hormones.

### Effect of LEAP-2 on energy homeostasis and thermogenesis in aged mice

The relationship between ghrelin and GHS-R in aging has been reported, with GHS-R exerting opposite effects on WAT and BAT during aging. *Ghsr*^−/−^ mice fed on regular chow have reduced fat mass and an improved lipid profile [34], highlighting the differential effects of ghrelin and GHS-R deficiency on body composition and energy homeostasis during aging [35]. We found increased circulating levels of LEAP-2 (Suppl. Figure 1d) and liver mRNA expression in aged animals (Suppl. Figure 1e).

**Fig. 5:**
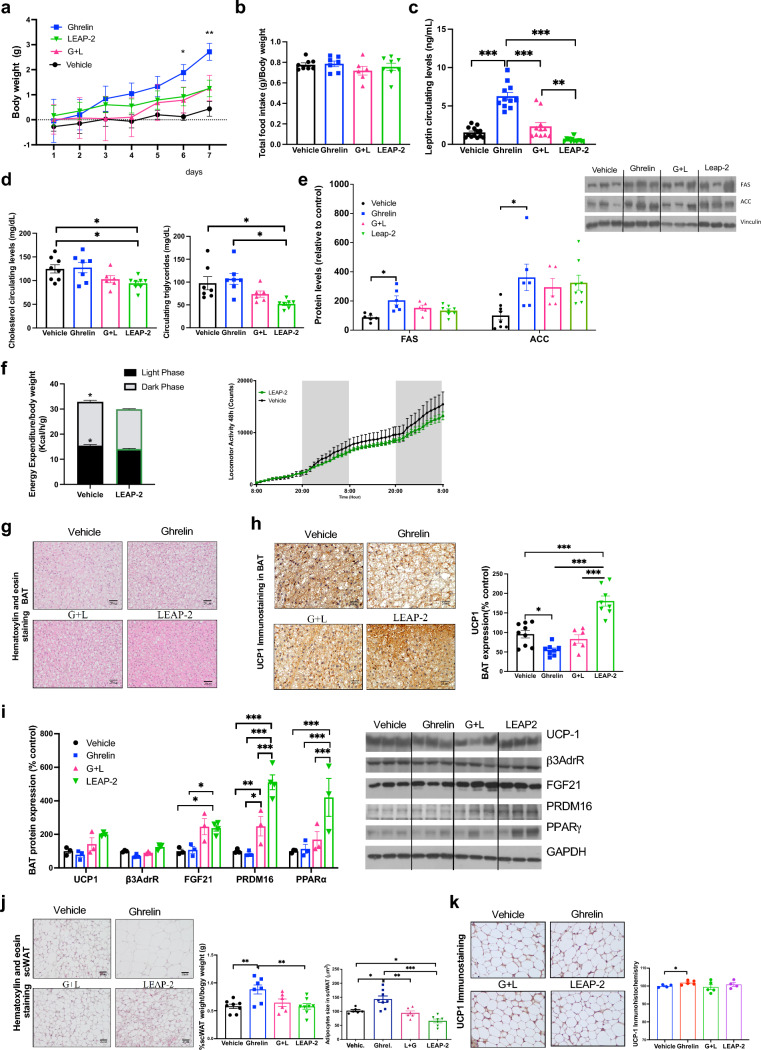
Central chronic LEAP-2, ghrelin, ghrelin and LEAP-2 or vehicle in aging mice maintain thermogenic effect. **a** Body weight change during treatment (7 days) in mice with 30 months age old. **b** Accumulated food intake in relation of body weight during all treatment. **c** Circulating levels of leptin in all groups after 7 days of treatment. **d** Circulating cholesterol and triglycerides levels after 7 days of treatment. **e** Quantification of immunoblot analysis of de novo lipogenesis proteins FAS and ACC in WAT and representative immunoblot after 7 days treatment. **f** Energy expenditure corrected by body weight and locomotor activity during 48 h in control and LEAP-2 treated mice. **g** Representative Hematoxylin & Eosin staining of BAT of treated mice after 7 days of treatment in aging mice. **h** Representative UCP1 immunohistochemistry and semiquantification of UCP1 of BAT after 7 days treatment. **i** Quantification of immunoblot analysis of thermogenesis proteins as UCP1, β3-adrenergic R, FGF21, PRDM16 and PPARγ in BAT and representative immunoblot. **j** Representative Hematoxylin & Eosin staining, weight and adipocyte size measurements of scWAT. **k** Representative UCP1 immunohistochemistry and semiquantification of UCP1 of scWAT. Data is expressed as mean ± SEM. *p ≤ 0.05, **p ≤ 0.01, ***p ≤ 0.001

Thereby, we decided to study the actions of LEAP-2 in aging mice. We chronically administered LEAP-2, ghrelin, ghrelin-LEAP-2 or vehicle intracerebroventricularly (icv) to 30-month-old mice. Compared to untreated control mice, LEAP-2 failed to produce changes in body weight (Fig. 5a), on the other hand, ghrelin administration led to a clear increase in body weight, which was blunted by co-administration of LEAP-2 (Fig. 5a). Both LEAP-2 and ghrelin failed to affect food intake (Fig. 5b). In aged mice, ghrelin treatment elevated circulating leptin levels, a marker of adiposity, while LEAP-2 administration decreased leptin levels. Co-administration of ghrelin and LEAP-2 effectively reversed the ghrelin-induced increase in leptin (Fig. 5c). This aligns with the observed reductions in circulating triglycerides and cholesterol compared to controls (Fig. 5d). Similarly, no significant effect of LEAP-2 was observed under basal or stimulated conditions on FAS or ACC (Fig. 5e).

Surprisingly, LEAP-2 treatment led to a slight decrease in energy expenditure (Fig. 5f), potentially contributing to the observed trend towards higher body weight compared to control mice. No changes in locomotor activity were observed (Fig. 5f). We assessed the potential effect of LEAP-2 in the BAT of aged animals. Histological analysis revealed smaller lipid droplets (Fig. 5g) and increased UCP1 immunolabelling (Fig. 5h). Protein levels of other thermogenic proteins, including PRDM16, PPARα, and FGF21 were elevated, with a clear trend for UCP1 (Fig. 5i), suggesting partial activation of the thermogenic program in LEAP-2-treated mice. Ghrelin administration resulted in an increase in adipocyte size and subcutaneous WAT weight (Fig. 5j). Administration of LEAP-2 alone or in combination with ghrelin significantly reduced subcutaneous WAT size under both basal and stimulated conditions. However, LEAP-2 treatment did not exhibit a significant effect on UCP1 levels under basal or stimulated conditions (Fig. 5k).

These findings suggest that in aged mice, central LEAP-2 administration counteracts ghrelin-induced weight gain by triggering thermogenic activity in BAT but does not induce browning of scWAT.

## Discussion

This study demonstrated that the brain infusion of LEAP-2 reduces body weight in young mice and blocks ghrelin-induced weight gain in both young and old mice fed a chow diet. However, this effect is lost in diet-induced obese rodents. The effect on body weight was associated with a decrease in adiposity, as assessed by measuring leptin levels, as well as a decrease in basal triglyceride levels and ghrelin-induced cholesterol levels. Furthermore, our data indicate that the effect of LEAP-2 on body weight is associated with an increase in energy expenditure (EE). Indeed, we provide evidence that its stimulatory effect EE could be mediated by the increased activation of BAT and/or increased browning of scWAT. Taken together, this effect of LEAP-2 on EE provides further support for its role as a robust regulator of energy homeostasis. Our data showing this effect of LEAP-2 at the central level resembles that previously reported [36] and is consistent with data showing that neuronal knock down of the GHSR1a HFD-induced weight gain by increasing EE [37]. Hence, we decided to study in depth its effects taking into consideration the environmental temperature at which the animals were exposed to, their diet and their age. All these factors are considered highly relevant when assessing the role of ghrelin in relation to the different components of energy homeostasis [22]. This is necessary to reach firm conclusions regarding the homeostatic role of LEAP-2 and its potential as a therapeutic agent [22, 38].

The importance of housing temperature in comparative studies involving rodents and humans regarding energy homeostasis has been firmly established [39–41]. The concept of thermoneutrality in humans spans a range from 19 ºC to 30 ºC, indicating that individuals in developed societies are rarely exposed to temperatures below this range. In contrast, mice, which are typically housed at 22 °C, must increase their energy expenditure by 30% to maintain core body temperature [42]. Therefore, any studies investigating mechanisms related to energy homeostasis may be influenced by this phenomenon, particularly when considering that the activation of BAT and browning of WAT are regarded as potential strategies for preventing and treating obesity [43–46].

To address this issue, we conducted a study to assess the impact of LEAP-2 in mice at thermoneutrality. Our findings, demonstrating that LEAP-2 increased energy expenditure in animals at 22 ºC, suggest that it was reversing the inhibitory effect of the ghrelin system on BAT activation or browning, as indicated by indirect measurements based on the expression of UCP1. Conversely, our data reveal that the effect of LEAP-2 under thermoneutral conditions was significantly diminished, implying that the ghrelin system plays a minor role in suppressing energy expenditure at 30 ºC. This aligns with the concept that ghrelin can be considered a survival hormone [47]. Therefore, when animals are exposed to lower temperatures, such as 22 ºC, ghrelin suppresses thermogenesis in BAT and browning of scWAT to conserve energy. However, once animals reach thermoneutrality, the effect of ghrelin is substantially reduced, explaining the lack of impact of LEAP-2 under these conditions.

A strong interrelationship between the ghrelin system and exposure to a HFD is well-established. Genetic silencing of either ghrelin [48] or its receptor GHSR1a [37] prevents HFD-induced weight gain in mice exposed early in life. Additionally, the orexigenic effect of exogenously administered ghrelin is blunted in animals exposed to HFD, while ghrelin-induced adiposity is maintained [21]. On the other hand, it has been recently reported that mice exposed to HFD exhibit higher intrahepatic LEAP-2 synthesis and elevated circulating levels compared to chow diet, indicating overexpression of LEAP-2 in states of nutritional excess [49]. Similar data was obtained in humans when compared normal versus obese subjects, both children and adults [16, 29, 50]. Consistent with these findings, genetic deletion of LEAP-2 in male mice fed a standard diet exposed to acute peripheral administration of ghrelin exhibits increased food intake and increased GH secretion, but does not affect body weight and weekly food intake in male mice exposed to HFD [51] indicating a state of ghrelin resistance that could affect agonists and antagonists of the GHSR1a. Our data showing that in a similar experimental setting, icv administration of LEAP-2 failed to influence weekly food intake or energy expenditure in animals exposed to HFD, indicates that the reason of this resistance is unrelated to impairment of ghrelin or LEAP-2’s ability to cross the blood–brain barrier. Whether this alteration is related to changes in the expression of the GHSR1a or its signaling pathway at the central level remains to be established. Previous data assessing the effect of exposure to HFD for one week showed that ghrelin-induced food intake but not adiposity was impaired. Our data, obtain from animals exposed to chronic HFD for a more extended duration (15 weeks) showed that long-term exposure leads to an impairment of ghrelin and LEAP-2 effects in both food intake and adiposity.

In summary, the main effects of both ghrelin and LEAP-2 in energy homeostasis are largely lost upon chronic exposure to HFD.

A significant hallmark of aging is the association with decreased circulating levels of GH and insulin-like growth factor 1 (IGF-1) [52, 53]. However, the question of whether circulating ghrelin levels are altered during the aging process and to what extent aging leads to a state of ghrelin resistance remains a topic of debate [54]. Consequently, there is considerable interest in assessing the response to LEAP-2 in aged mice. Our data revealed that the administration of LEAP-2 alone in aged mice failed to produce any substantial effects on food intake or body weight, suggesting the presence of either reduced ghrelinergic activity or a state of ghrelin resistance. Notably, our findings indicate that the orexigenic effect of ghrelin is lost in aged animals, pointing towards a state of ghrelin resistance. Given that we administrated ghrelin directly at the central level, this resistance seems to be independent of ghrelin's ability to traverse the blood–brain barrier. Conversely, our data also demonstrated that ghrelin was capable of increasing body weight and adiposity, as evidenced by elevated leptin levels providing further support for the existence of distinct neuronal pathways governing these two effects. Intriguingly, while the effects of ghrelin on leptin were reversed when LEAP-2 was co-administered, it failed to modify FAS and ACC protein levels. It's also important to note that our study did not observe any discernible effects of ghrelin and LEAP-2 in aged animals in terms of their impact on biomarkers of BAT activity. This lack of effect could be explained by recent findings showing that aged mice produced much fewer beige adipocytes from these cells than their younger counterparts. The mechanisms involved could be related to aging-induced impairment of de novo beige adipogenesis from adipocyte stem and progenitor cells which is due to dysregulation of unknown signaling pathways since this impairment occurs in vivo but no in vitro [55]. Future investigations involving a comprehensive assessment of various components within the ghrelin system are warranted before mechanistic conclusions can be drawn.

In summary, our data continue to bolster the significance of LEAP-2 as a pivotal component within the ghrelin system. We present compelling evidence that both corroborates and extends our understanding of its interaction with ghrelin concerning various facets of energy homeostasis, encompassing food intake, energy expenditure, and adiposity in mice subjected to a standard diet [14]. Furthermore, our study introduces fresh insights into the central effects of LEAP-2 within distinct experimental contexts, each possessing high translational relevance. These contexts encompass exposure to a high-fat diet, thermoneutrality, and the aging process, thereby expanding our comprehension of LEAP-2's multifaceted role in regulating energy balance.

### Supplementary Information

Below is the link to the electronic supplementary material.

Supplementary file1 Supplementary figure 1: Variations of circulating and hepatic levels of LEAP-2 with diet and aged. **a** Circulating levels of T4 and IGF-1 in mice with administration of LEAP-2, ghrelin, ghrelin and LEAP-2 or vehicle during 7 days. **b** Circulating levels of LEAP-2 in mice under HFD during 20 weeks. **c** Liver leap2 mRNA expression of mice under HFD during 20 weeks. **d** Circulating levels of LEAP-2 in mice with 30 months of age. **e** Liver leap2 mRNA expression of in mice with 30 months of age. Data is expressed as mean ± SEM *p ≤ 0.05, **p ≤ 0.01, ***p ≤ 0.001. (PDF 64 KB)
