# Supplementary figures and images for: The effects of ghrelin and LEAP-2 in energy homeostasis are modulated by thermoneutrality, high-fat diet and aging
Source: J Endocrinol Invest. 2024 Feb 9;47(8):2061–74. doi: 10.1007/s40618-024-02307-4 (PMC11266414; doi:10.1007/s40618-024-02307-4)

**a**

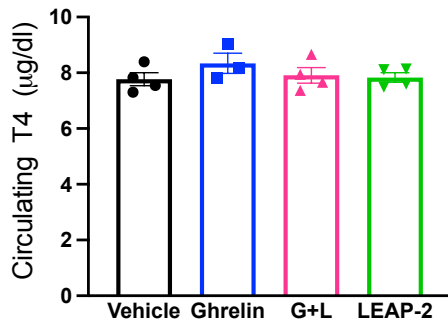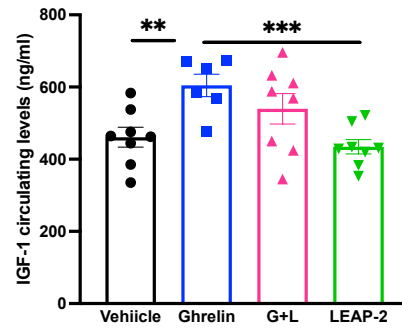

**b**

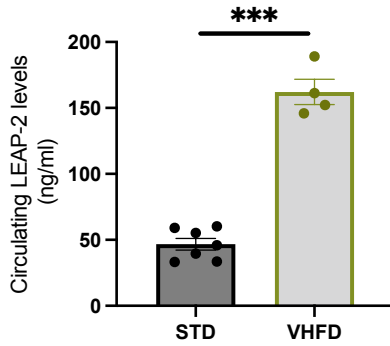

**c**

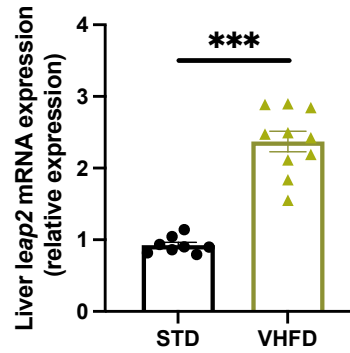

**d**

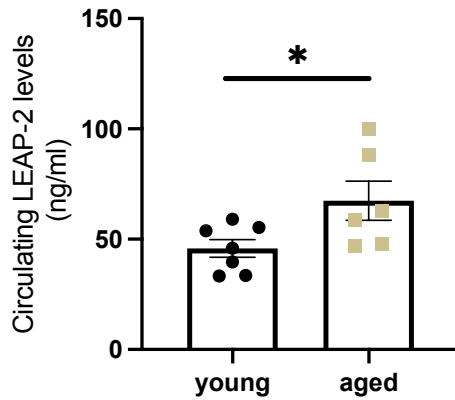

**e**

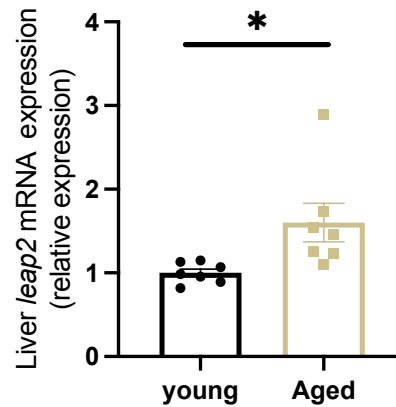

Supplement: Supplementary file 1 — Supplementary file1 Supplementary figure 1: Variations of circulating and hepatic levels of LEAP-2 with diet and aged. a Circulating levels of T4 and IGF-1 in mice with administration of LEAP-2, ghrelin, ghrelin and LEAP-2 or vehicle during 7 days. b Circulating levels of LEAP-2 in mice under HFD during 20 weeks. c Liver leap2 mRNA expression of mice under HFD during 20 weeks. d Circulating levels of LEAP-2 in mice with 30 months of age. e Liver leap2 mRNA expression of in mice with 30 months of age. Data is expressed as mean ± SEM *p ≤ 0.05, **p ≤ 0.01, ***p ≤ 0.001. (PDF 64 KB) [file 40618_2024_2307_MOESM1_ESM.pdf]
